# A novel method for measuring patients' adherence to insulin dosing guidelines: introducing indicators of adherence

**DOI:** 10.1186/1472-6947-8-55

**Published:** 2008-12-05

**Authors:** Massoud Toussi, Carine Choleau, Gérard Reach, Michel Cahané, Avner Bar-Hen, Alain Venot

**Affiliations:** 1 Laboratoire d'Informatique Médicale et Bioinformatique (LIM&BIO EA 3969), UFR SMBH, Université Paris 13, Bobigny, France; 2 Service d'Endocrinologie, Diabétologie et Maladies Métaboliques, Hôpital Avicenne, Assistance publique-Hôpitaux de Paris, et EA 3412, CRNH-IdF, Université Paris 13, Bobigny, France; 3 Association Aide aux Jeunes Diabétiques, Paris, France

## Abstract

**Background:**

Diabetic type 1 patients are often advised to use dose adjustment guidelines to calculate their doses of insulin. Conventional methods of measuring patients' adherence are not applicable to these cases, because insulin doses are not determined in advance. We propose a method and a number of indicators to measure patients' conformance to these insulin dosing guidelines.

**Methods:**

We used a database of logbooks of type 1 diabetic patients who participated in a summer camp. Patients used a guideline to calculate the doses of insulin lispro and glargine four times a day, and registered their injected doses in the database. We implemented the guideline in a computer system to calculate recommended doses. We then compared injected and recommended doses by using five indicators that we designed for this purpose: absolute agreement (AA): the two doses are the same; relative agreement (RA): there is a slight difference between them; extreme disagreement (ED): the administered and recommended doses are merely opposite; Under-treatment (UT) and over-treatment (OT): the injected dose is not enough or too high, respectively. We used weighted linear regression model to study the evolution of these indicators over time.

**Results:**

We analyzed 1656 insulin doses injected by 28 patients during a three weeks camp. Overall indicator rates were AA = 45%, RA = 30%, ED = 2%, UT = 26% and OT = 30%. The highest rate of absolute agreement is obtained for insulin glargine (AA = 70%). One patient with alarming behavior (AA = 29%, RA = 24% and ED = 8%) was detected. The monitoring of these indicators over time revealed a crescendo curve of adherence rate which fitted well in a weighted linear model (slope = 0.85, significance = 0.002). This shows an improvement in the quality of therapeutic decision-making of patients during the camp.

**Conclusion:**

Our method allowed the measurement of patients' adherence to their insulin adjustment guidelines. The indicators that we introduced were capable of providing quantitative data on the quality of patients' decision-making for the studied population as a whole, for each individual patient, for all injections, and for each time of injection separately. They can be implemented in monitoring systems to detect non-adherent patients.

## Background

In some diseases such as diabetes, patients manage their own therapy. Insulin dependent diabetic patients are often advised to use dose adjustment guidelines to calculate their doses of insulin for each self-administration. However, they may follow their guidelines totally, partially, or not at all. The adherence of patients to these guidelines is very important and can affect their blood glucose levels.

Methods for measuring patient adherence to drug therapy include self-reporting [1], patient interviews [2], pill counting [3], exploration of pharmacy records [4], measuring of active drug metabolites in the blood [5], and using electronic monitoring devices [6]. However, these methods are not applicable for the evaluation of patients' adherence to their insulin adjustment guidelines, because these guidelines do not determine correct insulin doses in advance. In fact, dose adjustment guidelines are distinct from common prescriptions by giving the patients the role of therapeutic decision-makers.

Diabetes is a major worldwide problem; and non-adherence to antidiabetic therapy and its impacts are well-documented [7]. Concerning insulin adjustment, the new technology allows the monitoring of patients through telecare systems, which store capillary blood glucose (CBG) and insulin doses [8]. Some systems even allow predicting of blood glucose based on lifestyle and therapeutic variables [9]. Although these systems have invaluable role in providing decision support and improving the quality of blood glucose control, they do not allow measuring the patients' conformance to insulin adjustment guidelines.

We present a method as well as a number of indicators that we developed for analyzing and monitoring of patients' adherence to their dose adjustment guidelines. We demonstrate our method in a group of type 1 diabetic patients who used an insulin dose adjustment guideline during supervised summer camps. We discuss the results and explain how our method and especially our indicators can be used in monitoring systems for real-time surveillance.

## Methods

### Patients

We used a database of logbooks of patients attending summer camps organized by Aide aux Jeunes Diabétiques (AJD). The latter is a non-profit organization with a major role in diabetes education in France. Eligible patients were those aged between 13 and 17, whose parents accepted their participation in the study. Non-eligible patients were those who did not manage their diabetes on their own, and those with a coexisting disease.

Twenty-eight patients (16 males and 12 females) were included in the study. They used a guideline to calculate the doses of their insulin injections four times a day. All patients had the same diet and the same kind of physical activity during the camp. Their daily carbohydrate intake was constant and fixed in advance for each meal by AJD nutritionists. The camp personnel supervised patients to assure their good dietary compliance. Physicians registered patients' CBGs, self-administered insulin doses, physical activities, and any event on their health status in an online database (the database is accessible to patients and their physicians under authentication [10]). The French Consultative Committee for Protection of Persons in Biomedical Research (CCPPRB) approved the research protocol and the guideline. The de-identified patient dataset is accessible as add-on material to this article. (Additional file 1). 

### The guideline

We used the official AJD guideline, taught to patients during summer camp. It consists of two pages of simple recommendations for the adjustment of insulin dose in a basal-bolus schema of insulin therapy including one injection of insulin lispro (or insulin aspart) before each meal, and one injection of insulin glargine before going to bed. This guideline was not specific for the kind of insulin, but for the schema of basal-bolus insulin therapy.

The guideline made use of patient's CBGs, injected doses of insulin, any advent of hypo- or hyperglycemia, and physical activity in a rule based algorithm composed of conditions and actions. It did not take into account carbohydrate intake because it was considered constant for each meal (breakfast, lunch, snack, and dinner) during the camp. As an example, to adjust the dose of insulin lispro before breakfast, the guideline proposed the patient to consider the CBG results obtained before lunch for two previous days, CBG measured at the moment of injection, and any physical activity over the next few hours. The guideline did not indicate the absolute dose of insulin. Instead, it provided instructions for the patients to adjust their own ongoing dose. The guideline is available as add-on material to this article. (Additional file 1).

### Computer methods

**Figure 1:**
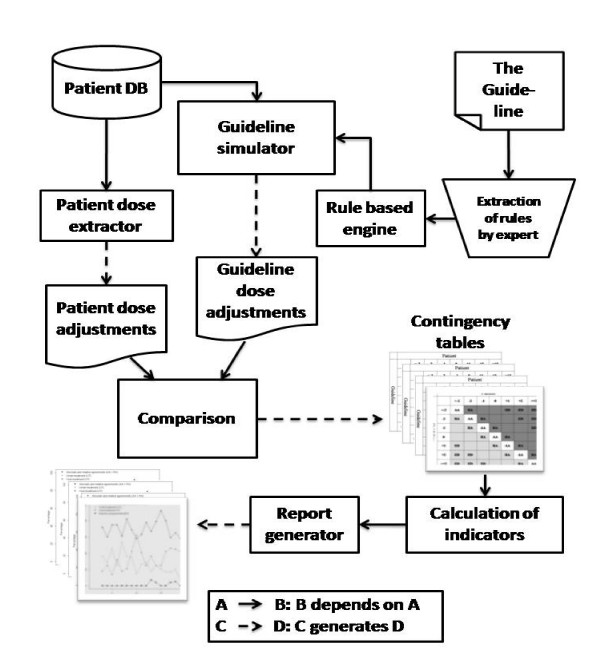
System architecture.

We extracted the rules of the guideline manually, and implemented them in a computer system, written in R programming language version 2.6.1 [11]. Our system uses data from patient logbooks to generate a dose adjustment for each insulin injection. It then compares these computer-generated dose adjustments with patient self-administered doses (figure 1). The system source code is available as add-on material to this article.

### Designing indicators

**Figure 2:**
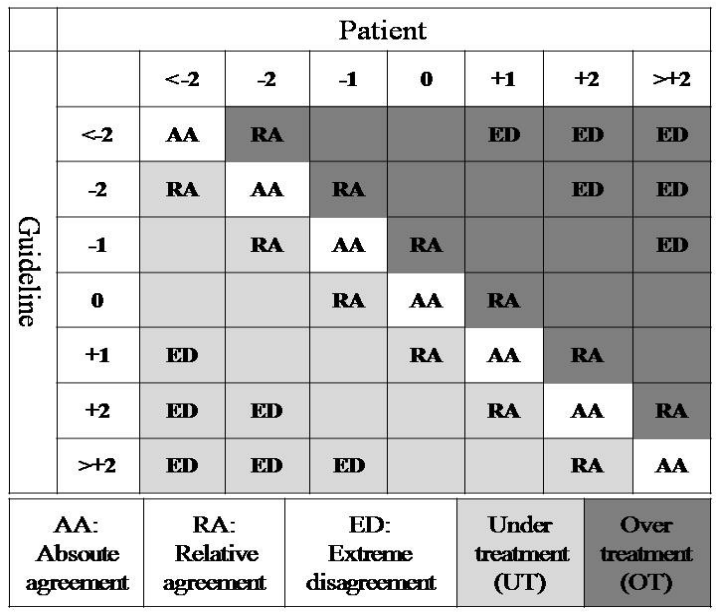
Indicators of adherence.

In order to be able to formalize and quantify the adherence, we categorized dose adjustments into seven discrete groups (table 1). We described each dose adjustment by variations with regard to the ongoing dose. By placing patient dose adjustments in columns and calculated dose adjustments in rows of a contingency table, we defined five indicators for quantifying patient's adherence to the guideline (figure 2).

**Table 1:** Dose adjustment categories.

**Symbol**	**Meaning**
**< -2**	Decrease dose by more than 2 units

**-2**	Decrease dose by between 1 and 2 units

**-1**	Decrease dose by between 0 and 1 units

**0**	Do not change the dose

**+1**	Increase dose by between 0 and 1 units

**+2**	Increase dose by between 1 and 2 units

**> +2**	Increase dose by more than 2 units

a) Absolute agreement (AA): On-diagonal cell counts, which correspond to injections for which the patient's dose adjustment is the same as generated dose adjustment.

b) Under-treatment (UT): Off-diagonal cell counts situated below the diagonal, which correspond to injections for which patient's dose adjustment is smaller than generated dose adjustment. Clinically, this indicator represents a hypoglycemia avoiding behavior.

c) Over-treatment (OT): Off-diagonal cell counts situated above the diagonal, which correspond to injections for which patient's dose adjustment is greater than generated dose adjustment. Clinically, this indicator represents a hyperglycemia avoiding behavior.

d) Relative agreement (RA): Off-diagonal cell counts adjacent to the diagonal, which correspond to adherent cases, but with some degree of tolerance.

e) Extreme disagreement (ED): The outermost off-diagonal cell counts, which correspond to "potentially dangerous" cases. In our study, we defined this area as cell counts where patient dose adjustment was in an opposite direction (i.e. increase versus decrease) of generated dose adjustment.

### Statistical methods

We used normal approximation of binomial distribution to construct confidence intervals (CI) for each indicator. We used also a weighted linear regression model [12] to determine the relationship between the time passed in the camp and each indicator. In the weighted analysis, we used the value of standard deviation of the indicator as the weight. Calculations are made by glm( ) function of R programming language version 2.6.1.

## Results

Patients mean age was 15 (SD = 1) years and their mean body mass index was 20.3 (SD = 2.8)kg/m^2^. Mean HbA_1C _was 7.8 (SD = 1.2)% at the beginning of the study. Mean CBG was 130 (SD = 70)mg/dl before breakfast, 170 (SD = 90)mg/dl before lunch, 180 (SD = 90)mg/dl before dinner, and 170 (SD = 90)mg/dl at bedtime.

Mean injected daily doses of insulin lispro were 9.5 (SD = 4.4) units before breakfast, 9.4 (SD = 5.3) units before lunch, and 8.6 (SD = 4.1) units before dinner. Mean injected daily dose of insulin glargine was 20.7 (SD = 7.2) units at bedtime.

Physicians made dose adjustments for patients on the first and the last two days of the camp. On all other days, patients adjusted themselves their doses of insulin under the supervision of physicians. Mean percentages of completion of the online database for dose adjustments before breakfast, lunch, dinner and at bedtime were respectively 92.6 (SD = 3.8), 90.4 (SD = 5.4), 86.7 (SD = 4.6) and 86.9 (SD = 4.8).

### Overall analysis

We analyzed 1656 pairs of dose adjustments (table 2). Two adjustments "do not change dose" and "decrease dose by up to 1 unit" were the most common adjustments in both patient (51% and 15% respectively) and computer-generated dose adjustments (62% and 20%). While guideline rules did not allow the dose to be adjusted by more than 2 units, patients made adjustments by more than 2 units in 7% of injections.

**Table 2:** Patients' versus computer-generated dose adjustments.

		**PATIENT DOSE ADJUSTMENTS**							
		**< -2**	**-2**	**-1**	**0**	**1**	**2**	**> +2**	**Total**

**GUIDELINE DOSE ADJUSTMENTS**	**< -2**	0	1	0	3	0	0	0	4
	
	**-2**	3	8	4	49	4	6	0	74
	
	**-1**	13	37	75	125	49	23	4	326
	
	**0**	31	69	143	609	101	51	28	1032
	
	**1**	12	14	23	49	46	29	15	188
	
	**2**	2	2	3	18	5	2	0	32
	
	**> +2**	0	0	0	0	0	0	0	0
	
	**Total**	61	131	248	853	205	111	47	1656

Overall rates of indicators for all patients and all insulin types were AA = 45% (CI = 2.3%), RA = 30%(CI = 2.1), ED = 2%, UT = 26%(CI = 2.3) and OT = 30%(CI = 2.1). As ED was too close to zero, its confidence interval was not calculated.

Although over-treatment (OT) was more common than under-treatment (UT), there were less cell counts of extreme disagreement (ED) in OT area than in UT area (10 and 16 cases respectively). This suggests that extreme disagreement was more likely to be a result of under-treatment, i.e. hypoglycemia-avoiding behavior.

### Injection related analysis

We calculated indicators separately for different injections (table 3). Patients were mostly adherent concerning insulin glargine with a rate of AA = 70% followed by insulin lispro before breakfast (AA = 38%). By considering RA cells also as adherent (with some tolerance), insulin lispro injections before dinner represented the highest level of adherence (78%).

**Table 3:** Indicator rates for each insulin injection.

**Injection**	**AA**	**RA**	**ED**	**UT**	**OT**	**N**
Lispro before breakfast	38	35	2	28	34	426

Lispro before lunch	37	36	2	33	30	419

Lispro before dinner	33	45	1	26	41	391

Glargine before sleep	70	6	2	15	15	420

N: number of injections.

### Patient related analysis

We calculated also the indicators for each patient (table 4). The highest score of adherence to the guideline was observed in a patient with AA plus RA ratio of 96%. On the other side, the highest rate of alarming non adherence -marked by the highest rate of ED indicator- was detected in a patient with an ED rate of 8%.

**Table 4:** Indicator rates for individual patients.

**Patient number**	**AA**	**RA**	**ED**	**UT**	**OT**	**N**
1	29	24	8	35	36	66

2	47	26	3	28	26	58

3	57	26	0	10	33	42

4	54	29	0	29	17	63

5	29	31	2	27	45	49

6	61	35	0	18	21	66

7	38	24	2	40	22	50

8	45	40	0	21	34	58

9	41	41	0	41	18	68

10	38	38	5	29	33	58

11	44	19	2	24	32	63

12	42	19	3	22	36	67

13	56	27	0	25	18	55

14	52	21	2	24	24	58

15	52	12	2	21	27	56

16	50	34	1	24	26	68

17	41	35	2	33	25	51

18	50	27	2	20	30	56

19	45	36	0	23	32	53

20	39	34	0	37	24	62

21	46	29	2	25	29	65

22	33	29	3	18	48	66

23	62	27	0	14	23	64

24	40	38	0	27	33	52

25	37	33	0	37	27	52

26	31	34	2	17	52	64

27	48	34	0	23	28	64

28	44	37	3	26	31	62

All	45	30	2	26	30	1656

N: number of injections.

### Time related analysis

**Figure 3:**
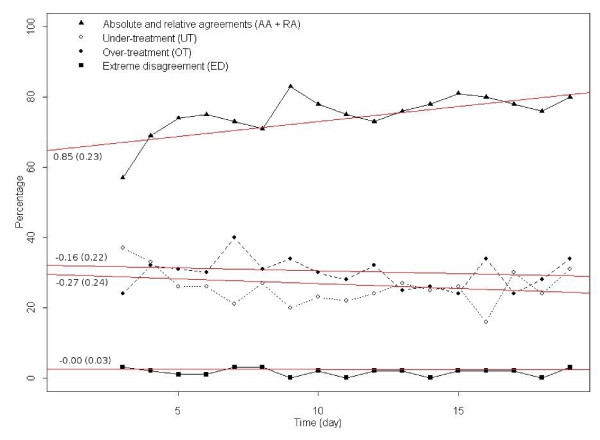
**Changes of indicators over time**. Coefficients: slope (on the left end of regression lines), standard errors (in parentheses).

We analyzed the evolution of patients' therapeutic decisions over time by tracing the indicators of each day separately (figure 3). The AA plus RA curve, which was more interesting for clinicians than AA or RA alone, showed an increasing trend during the camp. The ED curve showed fluctuations apparently due to different weekend activities and feasts (non-adherence to the guideline increased during festivities). OT and UT curves showed that over-treatment was more common than under-treatment, almost all time.

We fitted a weighted generalized linear model to examine the evolution of each indicator. The model fitted well (significance = 0.002) for the AA plus RA curve, but not for other indicators. It showed that the overall agreement progressed during the camp, and the rates of over- and under-treatments decreased smoothly. The extreme disagreement indicator, however, did not show a sensible change.

## Discussion

We presented a method for measuring and analyzing patients' adherence to an insulin dose adjustment guideline. A computer system deployed the guideline, and generated dose adjustments for each insulin injection. The system compared these dose adjustments with patient-calculated dose adjustments, using contingency tables and related indicators. We traced the fluctuations of indicators over time, and showed their significance by use of a weighted linear regression model.

By using indicators for measuring patients' adherence to guidelines, this study extends the method comparing insulin dose adjustments to computer-based insulin dose adjustment algorithms [13]. The implementation of insulin adjustment algorithms in a computer program and comparing generated results with patient-calculated results is already documented in another study, but without the use of indicators. Its authors reported an overall rate of adherence to the guideline of 89%. This adherence rate is comparable to the AA plus RA ratio (75%) obtained in our study. They did not deal with day-to-day changes in the adherence, or with particular types of patient behavior, such as under- or over-treatment [14].

We used a locked database in retrospective "offline" settings, but the same method with no change is actually applicable for an online database to monitor patients' adherence in real-time.

Three indicators, namely AA, OT, and UT, are based on statistically known concepts of on- and off-diagonal values of contingency tables. We designed two more indicators of RA and ED, which overlap with other indicators, for their clinical interest. The width of the zones defining these indicators on the contingency table depends on how precise the users of the method want to define their discrete categories. For example, if categories of dose adjustment were defined in two-unit intervals rather than one-unit intervals, the zone of relative agreement would be absorbed by that of absolute agreement (in this case, the categories would be < -2, -2, 0, +2, > +2, and the contingency table would have 25 cells instead of 49).

In our study, patient-calculated dose adjustments were in "absolute agreement" with those generated by the computer in less than half of injections (AA = 45%). We introduced the notion of "relative agreement" to detect those injections (RA = 30%) which are not strictly the same doses as calculated by the computer, but can be considered acceptable with a degree of tolerance of one unit. A similar strategy is used in the analysis of glucometer performances in form of the well-known Error Grid Analysis [9,15].

While measuring the impact of educational camps, it is especially interesting to know if there is an improvement in patients' adherence during the camp. In our study, this is confirmed by the crescendo curve of cumulative AA and RA indicators during the camp, which is demonstrated by a well-fitted linear model. However, for other indicators we could not fit a statistically significant model, which means that their predictive value for the studied population was limited.

The detection of alarming situations is of paramount importance in monitoring of patient's adherence. The extreme disagreement (ED) indicator is designed to satisfy this need. Although its overall trend is shown to be stable by the regression line, its peaks may necessitate intervention in a real-time monitoring system (figure 3).

Two indicators, UT and OT, explain at least partly what patients do when they do not follow the guideline, and why. For example, the frequency of OT is particularly high for a patient (table 4). This implies that this patient tends to try to avoid hyperglycemia by injecting a greater dose of insulin than what is recommended by the guideline. In a monitoring system using our indicators, this patient can be easily detected.

A limitation of our study is that the significance of our indicators depends on the number of observations. Therefore, we need an initial period of a few days before being able to monitor a patient's behavior. Another limitation is the guideline itself. In fact, in 2006, AJD used the same guideline for both patients under insulin lispro and patients under insulin aspart. As our study was non-interventional, we used the guideline which was actually taught to the studied population during 2006. However, the study on this guideline showed some of its shortcomings, and helped developing a new version of the guideline the following years.

Our proposed method and indicators are not specific to insulin self-administration. They can be applied in any other disease where patients decide for their dose of therapy, such as asthma or migraine headache.

## Conclusion

Our method allowed the analysis and monitoring of patients' adherence to an insulin adjustment guideline. The indicators were capable of providing quantitative data on the adherence of patients to the guideline not only for the studied population as a whole, but also for each individual patient. We are currently implementing these indicators in a telemedicine project, supported by the French National Authority for Health (FNAH), which aimed at monitoring young diabetic patients in real-time, and at alerting their non-adherent behavior to their physicians.

## Abbreviations

CBG: capillary blood glucose; FNAH: French National Authority for Health; AJD: Aide aux Jeunes Diabétique; SD: Standard deviation; CI: Confidence interval; AA: Absolute agreement; RA: Relative agreement; OT: Over treatment; UT: Under treatment; ED: Extreme disagreement.

## Competing interests

The authors declare that they have no competing interests.

## Authors' contributions

MT carried out computer methods, participated in data collection and preparation, participated in the design of indicators and their calculation, and drafted the manuscript. CC carried out the follow-up of patients, obtained authorization from ethics committee, and participated in data collection, preparation and validation. GR verified the clinical value of findings and delimited indicators based on their clinical interest. MC organized patients' observations in camps, and participated in clinical validation of findings. AB carried out statistical methods, participated in the design of indicators, and verified statistical findings. AV conceived of the study, and participated in its design and coordination and helped to draft the manuscript. All authors read and approved the final manuscript.

## Pre-publication history

The pre-publication history for this paper can be accessed here:



## Supplementary Material

Additional file 1 **Studied guidelines and system source code**. This is an html folder containing the studied guidelines in English and French, the de-identified patient database and the source code of the computer system. The file may be downloaded and unzipped in a folder. All the content of the folder can be accessed by double-clicking on "index.htm" file in the folder root. Click here for file
