# Supplementary material for: A novel method for measuring patients' adherence to insulin dosing guidelines: introducing indicators of adherence
Source: BMC Med Inform Decis Mak. 2008 Dec 5;8:55. doi: 10.1186/1472-6947-8-55 (PMC2636792; doi:10.1186/1472-6947-8-55)
Supplement: Additional file 1 — Studied guidelines and system source code. This is an html folder containing the studied guidelines in English and French, the de-identified patient database and the source code of the computer system. The file may be downloaded and unzipped in a folder. All the content of the folder can be accessed by double-clicking on "index.htm" file in the folder root. [file 1472-6947-8-55-S1.zip › guidelines and sourcecode/ajd_guidelie_lispro_glargine_en.pdf]

Rules of insulin dose adjustment for basal-bolus schema

-The rapid insulin of the morning is responsible for morning glycemia.

-The rapid insulin of the noon is responsible for after noon glycemia.

-The rapid insulin of the evening is responsible for evening glycemia.

-The lent insulin which is injected before dinner or before going to bed assures a "basal dose" of insulin for the following night and day.

1- Begin by adjusting the lent insulin: its dose is adjusted based on glycemia measured in distance of meals, especially the fasting glycemia in the morning:

-If you had an unexplained hypoglycemic faint during the previous night, or if your fasting glycemia was less than 60 mg/dl, decrease the dose of insulin glargine by up to 2 units.

-If your fasting glycemia was too high G>180mg/dl for several consecutive days (for example three days), increase the dose of insulin glargine by up to 2 units.

2- ***Recommendations for adjusting rapid insulin in the morning (injected before breakfast)***

-If you had an unexplained episode of hypoglycemia yesterday morning, or your glycemia before lunch yesterday was below 60 mg/dl, decrease your insulin lispro dose by up to half a unit for doses less than 5 units, by up to 1 unit for doses between 5 and 15 units, and by up to 2 units for doses of more than 15 units.

-If your glycemia before lunch was above 180 mg/dl during the last two days, increase your insulin lispro dose by up to half a unit for doses less than 5 units, by up to 1 unit for doses between 5 and 15 units, and by up to 2 units for doses of more than 15 units.

3- Recommendations for adjusting rapid insulin at noon (injected before lunch)

-If you had an unexplained episode of hypoglycemia yesterday after noon, or your glycemia before dinner yesterday was below 60 mg/dl, decrease your insulin lispro dose by up to half a unit for doses less than 5 units, by up to 1 unit for doses between 5 and 15 units, and by up to 2 units for doses of more than 15 units.

-If your glycemia before dinner was above 180 mg/dl during the last two days, increase your insulin lispro dose by up to half a unit for doses less than 5 units, by up to 1 unit for doses between 5 and 15 units, and by up to 2 units for doses of more than 15 units.

4- Recommendations for adjusting rapid insulin in the evening (injected before dinner)

-If you had an unexplained episode of hypoglycemia yesterday evening, or your glycemia before sleep yesterday was below 130 mg/dl, decrease your insulin lispro dose by up to half a unit for doses less than 5 units, by

up to 1 unit for doses between 5 and 15 units, and by up to 2 units for doses of more than 15 units.

-If your glycemia before sleep was above 180 mg/dl during the last two days, increase your insulin lispro dose by up to half a unit for doses less than 5 units, by up to 1 unit for doses between 5 and 15 units, and by up to 2 units for doses of more than 15 units.

5- If your glycemia is currently above 180 mg/dl, increase the insulin lispro dose that you are about to inject by up to 5% of the total number of insulin lispro units you inject daily, rounded to the closest integer.

6- If you are going to do, during the next hours, a physical activity to which your body is not used to, decrease the preceeding lispro insulin dose that you are about to inject by up to 20%.

ATTENTION : PERSONALIZE THE RULES IF BASED ON YOUR OBTAINED RESULTS
