# Supplementary material for: A novel method for measuring patients' adherence to insulin dosing guidelines: introducing indicators of adherence
Source: BMC Med Inform Decis Mak. 2008 Dec 5;8:55. doi: 10.1186/1472-6947-8-55 (PMC2636792; doi:10.1186/1472-6947-8-55)
Supplement: Additional file 1 — Studied guidelines and system source code. This is an html folder containing the studied guidelines in English and French, the de-identified patient database and the source code of the computer system. The file may be downloaded and unzipped in a folder. All the content of the folder can be accessed by double-clicking on "index.htm" file in the folder root. [file 1472-6947-8-55-S1.zip › guidelines and sourcecode/ajd_guidelie_lispro_glargine_fr.pdf]

Règles d'adaptation des doses d'insuline dans le schéma :
Analogue rapide, Analogue rapide, Analogue rapide, Lantus.

- L'insuline rapide du matin est responsable des glycémies de la matinée.
- L'insuline rapide du midi est responsable des glycémies de l'après-midi.
- L'insuline rapide du soir injectée avant le dîner est responsable des glycémies de la soirée.
- L'insuline Lantus injectée avant le dîner ou au coucher assure un «débit de base » d'insuline toute la journée et toute la nuit.

1- Commencer par régler la Lantus : on règle sa dose en regardant les glycémies à distance des repas, notamment le matin au réveil :

- Si il y a eu dans la nuit de la veille un malaise hypoglycémique inexpliqué, ou si la glycémie du réveil a été inférieure à 0.60 g/l, baisser de 2 unités la Lantus.

- Si la glycémie du matin au réveil est trop élevé G > 1.80g/l plusieurs jours de suite (trois par exemple), augmenter de 2 unités la Lantus.

2- Pour régler l'insuline rapide du matin (injectée avant le petit-déjeuner) :

- Si il y a eu dans la matinée de la veille un malaise hypoglycémique inexpliqué, ou si la glycémie avant le déjeuner a été inférieure à 0.60 g/l, baisser l'insuline rapide du matin de 1/2 unité si la dose est inférieure à 5unités, de 1unité si la dose est comprise entre 5 et 15 unités, de 2 unités si la dose est supérieure à 15 unités.

- Si la glycémie avant le déjeuner a été supérieure à 1.80 g/l deux jours de suite, augmenter l'insuline rapide du matin de 1/2 unité si la dose est inférieure à 5unités, de 1unité si la dose est comprise entre 5 et 15 unités, de 2 unités si la dose est supérieure à 15 unités.

3- Pour régler l'insuline rapide du midi (injectée avant le déjeuner) :

- Si il y a eu dans l'après-midi de la veille un malaise hypoglycémique inexpliquée, ou si la glycémie avant le dîner a été inférieure à 0.60 g/l, baisser l'insuline rapide du midi de 1/2 unité si la dose est inférieure à 5unités, de 1unité si la dose est comprise entre 5 et 15 unités, de 2 unités si la dose est supérieure à 15 unités.

- Si la glycémie avant le dîner a été supérieure à 1.80 g/l deux jours de suite, augmenter l'insuline rapide du midi de 1/2 unité si la dose est inférieure à 5unités, de 1unité si la dose est comprise entre 5 et 15 unités, de 2 unités si la dose est supérieure à 15 unités.

4- Pour régler l'insuline rapide du soir (injectée avant le dîner) :

- Si il y a eu dans la soirée la veille un malaise hypoglycémique inexpliquée, ou si la glycémie avant le coucher a été inférieure à 1.30 g/l, baisser l'insuline rapide du soir de 1/2 unité si la dose est inférieure à 5unités, de 1unité si la dose est comprise entre 5 et 15 unités, de 2 unités si la dose est supérieure à 15 unités.

- Si la glycémie avant le coucher a été supérieure à 1.80 g/l deux jours de suite, augmenter l'insuline rapide du soir de 1/2 unité si la dose est inférieure à 5unités, de 1unité si la dose est comprise entre 5 et 15 unités, de 2 unités si la dose est supérieure à 15 unités.

5- Si au moment de faire l'injection d'insuline le matin, ou le midi, ou le soir, la glycémie est supérieure à 1.80 g/l, on peut faire un supplément, par rapport à ce qui est prévu, de 5% de la dose totale d'insuline rapide de la journée en arrondissant au chiffre entier le plus proche.

6- Si on s'apprête à avoir une activité physique inhabituelle, il faut diminuer la dose d'insuline rapide qui précède de 20%.

ATTENTION : PERSONNALISER LES REGLES SI LES RESULTATS L'IMPOSENT
