# Supplementary material for: A novel method for measuring patients' adherence to insulin dosing guidelines: introducing indicators of adherence
Source: BMC Med Inform Decis Mak. 2008 Dec 5;8:55. doi: 10.1186/1472-6947-8-55 (PMC2636792; doi:10.1186/1472-6947-8-55)
Supplement: Additional file 1 — Studied guidelines and system source code. This is an html folder containing the studied guidelines in English and French, the de-identified patient database and the source code of the computer system. The file may be downloaded and unzipped in a folder. All the content of the folder can be accessed by double-clicking on "index.htm" file in the folder root. [file 1472-6947-8-55-S1.zip › guidelines and sourcecode/index.htm]

Indicators for monitoring patient adherence to dose adjustment
guidelines


#### A novel method for measuring patients� adherence to insulin dosing guidelines : introducing indicators of adherence

#### Massoud Toussi [1] , Carine Choleau [2,3] , G�rard Reach [2] , Michel Cahan� [3] , Avner Bar-Hen [1] , and Alain Venot [1]

#### (1) Laboratoire d�Informatique M�dicale et Bioinformatique (LIM&BIO EA 3869), UFR SMBH, Universit� Paris 13 ; (2) D�partement d�Endocrinologie, H�pital Avicenne, Assistance publique-H�pitaux de Paris ; and (3) Aide aux Jeunes Diab�tiques, Paris, France

---

## Annexes to the Article

- To download the Guideline of
  the Aide aux Jeunes Diab�tiques for the adjustment
  of insulin doses in a basal-bolus
  schema in English click here.
  For the French version click here.
- The source code of the computer system in R Programming Language which implements the above guidelines and generates
  dose adjustments, contingency
  tables, and graphics (patient records are also included in the
  package) is available
  for dowload here.

Thank you for visiting.

Your comments are welcome: massoudtoussi@gmail.com
